# Enhanced Performance for Multi-Forearm Movement Decoding Using Hybrid IMU–sEMG Interface

**DOI:** 10.3389/fnbot.2019.00043

**Published:** 2019-07-03

**Authors:** Waseem Shahzad, Yasar Ayaz, Muhammad Jawad Khan, Noman Naseer, Mushtaq Khan

**Affiliations:** ^1^Department of Robotics and Intelligent Machine Engineering, School of Mechanical and Manufacturing Engineering, National University of Sciences and Technology (NUST), Islamabad, Pakistan; ^2^National Center of Artificial Intelligence, Islamabad, Pakistan; ^3^Department of Mechatronics Engineering, Air University, Islamabad, Pakistan

**Keywords:** surface electromyography, pattern recognition, inertial measurement units, support vector machine, linear discriminant analysis

## Abstract

Control of active prosthetic hands using surface electromyography (sEMG) signals is an active research area; despite the advances in sEMG pattern recognition and classification techniques, none of the commercially available prosthetic hands provide the user with an intuitive control. One of the major reasons for this disparity between academia and industry is the variation of sEMG signals in a dynamic environment as opposed to the controlled laboratory conditions. This research investigated the effects of sEMG signal variation on the performance of a hand motion classifier due to arm position variation and also explored the effect of static position and dynamic movement strategies for classifier training. A wearable system is used to measure the electrical activity of the muscles and the position of the forearm while performing six classes of hand motions. The system is made position aware (POS) using inertial measurement units (IMUs) for different arm movement gestures. The hand gestures are decoded under both static and dynamic forearm movements. Four time domain (TD) features are extracted from the sEMG signals along with IMU-based arm position information. The features are trained and tested using linear discriminant analysis (LDA) and support vector machine (SVM) for both TD and TD-POS features. The results for the SVM show a significant difference between the static and dynamic approaches, while the TD-POS features show enhanced classification performance in comparison to the TD-based classification. Results have shown the effectiveness of the dynamic training approach and sensor fusion techniques to improve the performance of existing stand-alone sEMG-based prosthetic control systems.

## Introduction

The control of a dexterous upper limb using surface electromyography (sEMG) is an active research area since the 1960s. Mostly, the researchers have focused on the intuitive control of a multi-degree of freedom (DOF) prosthetic hand. Despite the advancements in the detection of the activity and the processing of the sEMG signals, most available commercial prostheses still utilize a pair of electrodes to control a single or multiple DOF of the prostheses (Parker and Scott, 1986). The current state-of-the-art myoelectric controllers require the amputee to switch between different pre-defined hand gestures by generating a pre-trained sequence of pulses (Farina and Aszmann, 2014). Such non-intuitive control is one of the major reasons for amputees to abstain from achieving a complete control of the prosthetic device (Engdahl et al., 2015; Chadwell et al., 2016). The stochastic nature and several extrinsic and intrinsic factors have affected the characteristics of the sEMG signals, and these causes have led to a major difficulty in the realization of intuitive prostheses control. In the past, the primary research focus was on the development of pattern recognition (PR) and classification techniques for the detection of different hand motion classes. As a result, a number of techniques including fuzzy systems (Lam et al., 2002), neural networks (Soares et al., 2003), spiking neural networks (Behrenbeck et al., 2019), fuzzy support vector machines (SVMs) (Xie et al., 2015), hidden Markov models (Chiang et al., 2008), and principal component analysis (PCA) (Naik et al., 2016) have shown high accuracy for hand movement decoding.

Although PR techniques have been successfully applied to prostheses control (Zecca et al., 2002; Khokhar et al., 2010; Suberbiola et al., 2013; Hargrove et al., 2017), the practical application of these techniques in the rehabilitation industry is very limited (Castellini et al., 2014). One possible reason for this gap between academic research (Jiang et al., 2012; Cordella et al., 2016) and the clinical acceptance of dexterous EMG-based prostheses control is due to the constrained laboratory conditions in which the sEMG signals are gathered from a test subject under static environment while ignoring the dynamic daily life scenarios (Chadwell et al., 2016). The unintended variation in sEMG signals occur due to movement artifacts (De Luca et al., 2010), variations in electrode placement (Hargrove et al., 2008), interelectrode spacing (De Luca et al., 2012), limb position (Radmand et al., 2014), muscle fatigue (Tkach et al., 2010), muscle length, and arm moments (Nourbakhsh and Kukulka, 2004). These unintended variations affect classifier performance by creating conditions that may not be encountered in a static laboratory environment or in clinical training sessions (Vujaklija et al., 2017). Since supervised learning is needed for most of the PR-based techniques for the decoding of hand movements, any significant variation of the sEMG characteristics negatively impacts the pre-trained classifier accuracy. Consider, for example, the variation of sEMG signals with the arm movement, since daily life activities are highly diverse and dexterous, a classifier trained with sEMG data acquired at a particular arm position or a set of positions will exhibit degradation as the arm is dynamically moved through different positions (Betthauser et al., 2018).

Fougner et al. (2011) have reported the effects of variation in arm movements on classifier performance. The research reported that the multiposition classifier training reduces the classification error from 18 to 5.7%. Classifier performance was further improved by adding the arm position information to the classifier through a sensor fusion of accelerometers and sEMG sensors. Chen et al. (2011) also reported similar results. Multiposition classifier training and the fusion of sEMG and accelerometer were reported for achieving 93% classification accuracy (Yu et al., 2018). The adverse effects of arm position variation in people with transradial amputation were studied (Geng et al., 2012) by comparing the characteristics of the sEMG signals of the intact limb and the amputated limb. The authors reported a less pronounced variation in the amputated limb as compared to the intact limb and suggested a sensor fusion of accelerometer mechanomyography (ACC-MMG) and sEMG to improve classifier performance. These previous studies have shown that the classifier performance can be improved after being trained and tested at a limited number of discrete positions. However, increasing the number of discrete training positions (more than five) decreases the classifier performance (Radmand et al., 2014). It is, thus, rational to assume that classifier performance may further degrade under dynamic forearm movements due to the increased number of possible positions.

In this research, we investigated the effects of arm position variation on the performance of hand motion classifiers at discrete static positions and under dynamic forearm movements. The objective was to suggest more realistic and pragmatic training procedures for multi-forearm movement classifier training. Multichannel muscle activation data, acquired through sEMG electrodes and arm position, measured using inertial measurement units (IMUs), were combined to study the effectiveness of sensor fusion techniques for classifier performance enhancement. Results for the dynamic forearm movement classifier showed better classification accuracy as compared to the static position classifier. The time domain (TD)-position aware (POS) feature-based classifiers performed significantly better than the TD feature-based classifiers, thus proving the effectiveness of sensor fusion techniques for rehabilitation.

## Materials and Methods

### Subjects

Ten healthy male subjects aged 19–43 (30.2 ± 2.5%) participated in the experiment. The experiments were conducted in accordance with the latest Declaration of Helsinki. The experiments were duly approved by the ethical committee of the National University of Sciences and Technology. Written consent was taken from the subjects prior to the start. None of the recruited participants had any history of muscular disorders.

### Data Acquisition System

**Figure 1:**
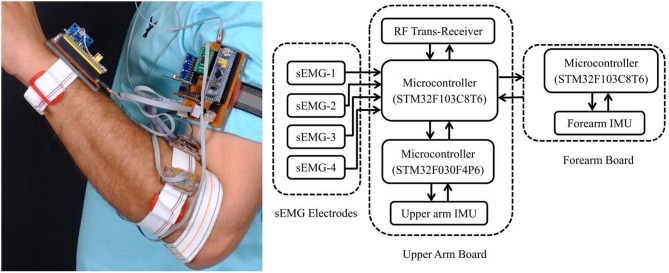
The wearable sensor system and its schematic diagram.

A fully independent wearable system (Figure 1) was designed to study the variation of the sEMG signals with the arm position. The design was kept modular to ensure subject comfort and ease of use. The system consisted of multiple sEMG sensors and IMUs to measure the electrical activity of the muscles and the position of the forearm relative to the shoulder.

The arm position was measured using a pair of IMUs (TDKs InvenSense MPU-9250) attached to the upper arm and forearm. The forearm IMU was placed proximal to the wrist, and the upper arm IMU was paced over the biceps brachii muscle. Each IMU consisted of a three-axis accelerometer, gyroscope, and magnetometer. The sensor data from each IMU was integrated by a dedicated microcontroller to measure the orientation of each arm segment in the form of a unit quaternion using the algorithms proposed by Madgwick et al. (2011).

**Figure 2:**
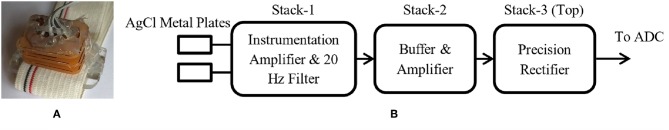
Stacked sEMG electrode **(A)** and its architecture **(B)**.

A set of four custom-designed dry electrodes were used to measure the muscular activity of the forearm (flexor and extensor muscles). Each electrode consisted of a pair of AgCl metal plates (5 × 10 mm) with an interelectrode spacing of 20 mm as per recommendations of SENIAM (surface EMG for the non-invasive assessment of muscles) (Hermie et al., 2000). Since sEMG signals are bipotential electrical signals and their magnitude varies from a few microvolts to several millivolts, they were processed for further use in PR and prosthesis control. The signals were amplified, filtered, and rectified. To ensure modularity and reduce the overall size, the amplification, filtering, and rectification circuits were stacked on top of the electrodes (Figure 2). This approach minimized cable motion artifacts and secured the system against power line noise.

Two of the four electrodes were placed on the forearm flexors, and the remaining two were placed at the forearm extensors ~2 cm from the elbow. A single reference electrode was placed on the elbow. No particular skin preparation techniques were used; however, a thin layer of conductive gel was applied before the start of each data acquisition session. The electrodes were held in position by an elastic band to ensure a reliable skin contact. Although increasing the number of sEMG electrodes can increase the classification accuracy, however, a previous study has shown that more than 90% accuracy is possible with careful placement of a minimum of three electrodes (Hargrove et al., 2007). More recently, (Benatti et al., 2015) has reported a 2% decrease in classifier accuracy as the number of electrodes are reduced from eight to four. Another important advantage of channel reduction is the increased system robustness against the sEMG signal corruption and degradation associated with shifting of electrodes during experiments and in actual prosthetic devices (Hargrove et al., 2008).

The analog signals from the electrodes were digitized by a microcontroller (STM32F103C8T6)-based data acquisition system having a 12-bit ADC. Since sEMG signals have 95% power spectrum in the 20–500 Hz range; therefore, a sampling frequency of 1 kHz was used based on a previous research (Ives and Wigglesworth, 2003). The four-channel sEMG data along with the two quaternions were wirelessly transmitted to a personal computer. Custom software, designed in Labview (Supplementary Figure 1), was used to bandpass-filter the raw signals with cutoff frequencies in the range of 20–500 Hz with no 50-Hz notch filtering. The software also calculated the position of the hand with reference to the shoulder using simple quaternion algebra. The filtered signals along with position information were monitored in real time, and the information was logged in CSV (comma-separated values) format for further processing and analysis.

### Data Acquisition Experiments

**Figure 3:**
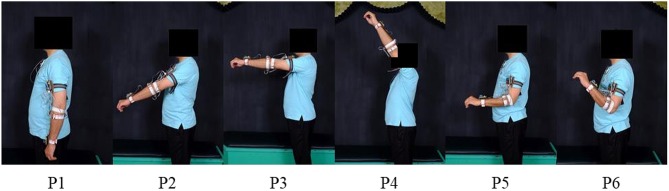
Six static forearm positions for the discrete position data. (Written informed consent was obtained for the publication of this image).

**Figure 4:**
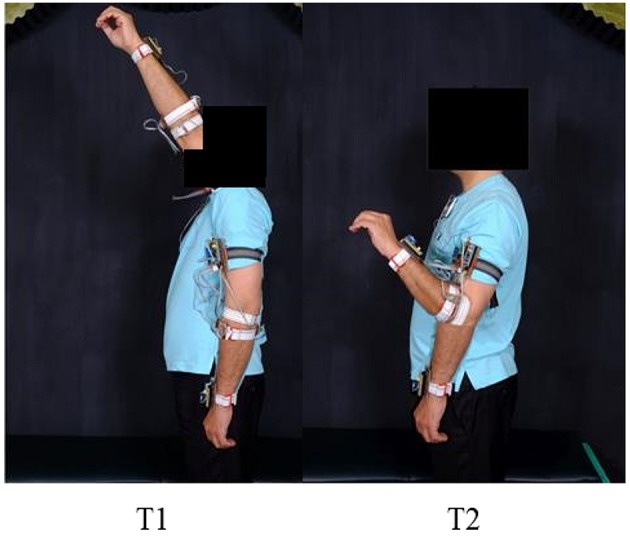
Illustration of the two trajectories for the continuous position data. (Written informed consent was obtained for the publication of this image).

Experimental data pertaining to six classes of hand motions including relaxed (RE), cylindrical (CY), lateral (LA), pinch (PI), open (OP), and spherical (SP) were collected from subjects for both discrete static and dynamic movements along the defined trajectories. The discrete static positions (P1–P6), illustrated in Figure 3, were chosen based on previous researches (Chen et al., 2011; Fougner et al., 2011; Khushaba et al., 2014). The trajectories for the dynamic movement, illustrated in Figure 4, were selected such as to adequately cover the static positions in a continuous movement. The data from each subject were gathered in several sessions. However, to ensure consistency, the complete data for a single class for the static positions and the dynamic movement were sampled in a single session. Each data acquisition session consisted of forearm movement along each of the two trajectories while executing the intended motion class. The motion along each trajectory was repeated six times, resulting in a total of 12 data files for a single class. The data for the discrete static positions involved six repetitions of the desired class at each of the positions (P1–P6), resulting in a total of 36 individual data files for each class. Each static repetition was sustained for 7–9 s with a gap of at least 10–15 s for subject comfort and fatigue prevention. Wherever applicable, class validity and uniformity across subjects were ensured by grasping a real object suitable for a particular class with moderate force.

### Feature Extraction and Classification

sEMG signal processing involves segmentation, feature extraction, PR, and classification. Segmentation is the process of subdividing the otherwise continuous data in windows of suitable lengths for feature extraction. In this study, a Labview based software (Supplementary Figure 2) was designed for data segmentation using a sliding window technique with a window length of 250 ms and an overlap of 200 ms, resulting in a new analysis window every 50 ms (Smith et al., 2011). Four TD features including average rectified value (ARV), slope sign changes (SSC), waveform length (WL), and integral sEMG (Iemg) were calculated for each window. The TD features were chosen because of their wide acceptance and computational efficiency (Hudgins et al., 1993; Scheme and Englehart, 2011). The four TD features concatenated with the position information (TD-POS) resulted in a feature vector represented by:

(1) $$\begin{array}{l} \left[\begin{array}{c} \;\left\{{\text{TD}}_{m,n}\right\}{|}_{m\;=\;1\ldots .4,\;n\;=\;1\ldots .4}\\ \;\left\{{\text{POS}}_{k}\right\}{|}_{k\;=\;x,y,z} \end{array}\right]\\ where\;m\;=feature\;no.\;\\ \;n\;=electrode\;no.\; \end{array}$$

The total discrete position data of each hand motion class were formed by combining the processed data of all the trials at each of the static positions. The total dynamic movement data of each class was formed by combining the processed data of all trials for both the trajectories. The data of each class were split in to two equal training and testing data sets through random sampling. The randomly split training data sets of all motion classes were then combined to form a single training data set (Supplementary Figure 3). The testing data set which was totally independent of the training data was also formed in a similar manner.

The training data set was used to train linear discriminant analysis (LDA) and SVM classifiers. The LDA is a classifier that has been extensively applied to multiclass problems because of its high performance at low computational cost (Englehart et al., 2003; Fougner et al., 2011; Yu et al., 2018). The SVM is a kernel-based classification technique that has been successfully applied to machine learning problems with linear and non-linear class boundaries. Previous studies by Oskoei and Hu (2008) applied the SVM classifier to sEMG data for gesture recognition and prosthesis control and reported the SVM outperforming LDA and multilayer perception (MLP) neural network-based classifiers. Betthauser et al. (2018) compared LDA, SVM, non-linear logistic regression (NLR), and MLP for classifying sEMG data and reported the SVM classifier achieving the best classification accuracy though at a higher computational cost. In this study, an SVM classifier with a radial basis function (RBF) kernel has been utilized. The code was implemented in R language (R Core Team, 2018) using package MASS (Venables and Ripley, 2002) for LDA and package e1071 (Meyer et al., 2019) for SVM. The parameters C and γ of the RBF kernel were optimized using grid search algorithm with a 10-fold cross-validation scheme. The testing and training of the classifiers were carried out individually for each subject, and no cross-subject training and testing were performed.

The effect of arm position variation on classifier performance and a comparison of the discrete and continuous position training approach were evaluated by training and testing both the classifiers (LDA and SVM) for the following different training and testing scenarios:

1. Classifiers trained and tested with only TD features of the discrete static positions data.
2. Classifiers trained with combined TD features and position information (TD-POS) for the discrete static positions.
3. Classifiers trained with only TD features of dynamic arm movement data.
4. Classifiers trained with the TD-POS of the dynamic arm movement data.
5. Six position-specific classifiers trained at only one of the discrete positions and tested against all the positions.

Unless specified, all the results are reported as average accuracy along with the standard error. The statistical comparison of classifiers was based on *t*-test, and the level of significance was set to *p* < 0.05.

## Results

### Discrete Position TD Features-Based Classifiers

**Figure 5:**
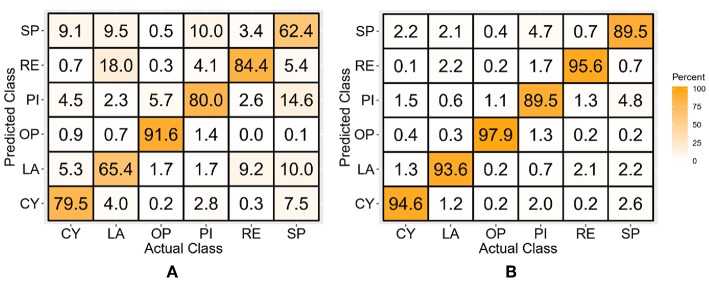
Confusion matrices for the TD features-based LDA **(A)** and SVM **(B)** classifier-trained with the discrete position data.

After the classifiers were trained and tested with only the TD features of the discrete position data, the average classification accuracy across all subjects and classes for the LDA classifier was 77.2 ± 4.6%, while for the SVM classifier it was 93.4 ± 1.4%, for which the confusion matrices are shown in Figure 5. The values across the diagonal indicate the percentage of samples correctly classified for each class, while those along the columns indicate the percentage of samples incorrectly classified as other classes. As an example, the left-most column of Figure 5B, which corresponds to the CY class, indicates that 94.6% of the test samples were correctly classified as CY, 1.3% were misclassified as LA, 0.4% as OP, 1.5% as PI, 0.1% as RE, and 2.2% as SP.

### Discrete Position TD-POS Features-Based Classifiers

**Figure 6:**
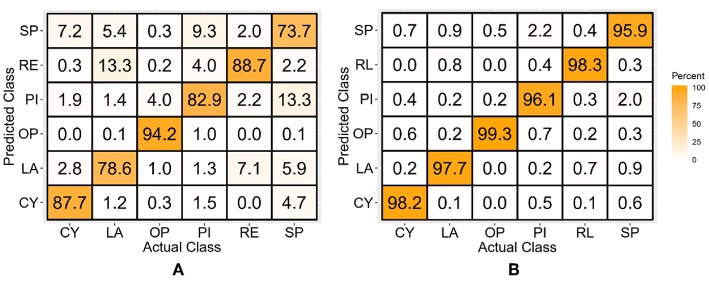
Confusion matrices of the LDA **(A)** and SVM **(B)** classifier trained with the TD-POS features of the discrete position data.

The LDA and SVM classifiers trained with the combined TD features and discrete position data showed average classification accuracies of 84.3 ± 3.0 and 97.5 ± 0.5%, respectively, averaged across all subjects and classes. The class-wise performance is evident from the confusion matrices shown in Figure 6. It is evident from the results that the position aware LDA classifier showed an average improvement of 6.1%, while the SVM classifier showed an average improvement of 4.1% over the TD features-only classifier.

### Continuous Position TD Features-Based Classifiers

**Figure 7:**
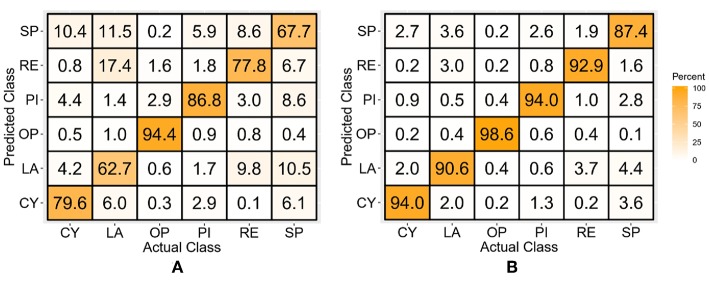
Confusion matrices of the LDA **(A)** and SVM **(B)** classifiers trained with TD features of continuous position data.

The classifiers trained with the TD features of the dynamic forearm movement data resulted in average classification accuracies of 78.2 ± 4.8 and 92.9 ± 1.5% for the LDA and SVM classifiers, respectively. The confusion matrices for both the classifiers are shown in Figure 7. It is interesting to note that there is no significant performance difference between the TD feature-based classifiers for the discrete and dynamic movement data (*p* > 0.05).

### Continuous Position TD-POS Features-Based Classifiers

**Figure 8:**
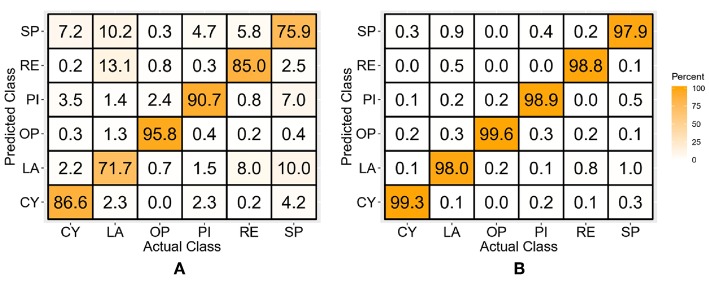
Confusion matrices for the TD-POS features-based LDA **(A)** and SVM **(B)** classifier trained with continuous position data.

Figure 8 shows the confusion matrices for the classifiers trained with the concatenated TD features and position data for the dynamic movement data set. The classification accuracy averaged across all subjects and classes is 84.3 ± 3.7% for the LDA and 98.7 ± 0.3% for the SVM classifier. This shows an average improvement of 5.8% over the continuous position TD feature-based classifier and an improvement of 1.2% over the discrete data TD-POS classifier.

### Position-Specific Classifiers

**Figure 9:**
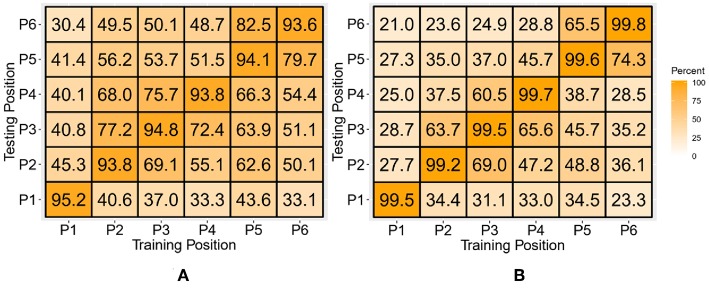
Interposition and intraposition classification accuracy matrices for LDA **(A)** and SVM **(B)** classifiers. The columns indicate the training positions, and rows indicate the testing positions for the classifiers.

The position-specific classifiers (LDA and SVM) were trained with the TD features of the discrete position data for each of the six static positions and tested individually against all the positions. The resulting confusion matrix is shown in Figure 9. The values across the diagonal indicate the intraposition classification accuracy averaged across all subjects and classes. The average intraposition classification accuracy for the SVM was 99.5 ± 0.08% and for the LDA was 94.2 ± 0.26%. The SVM performed significantly (*p* < 0.05) better than the LDA classifier.

## Discussion

The LDA classifier trained with the TD features of the continuous motion training data resulted in average classification accuracy (78.2%) as compared to the classifier trained with the TD features of the discrete position training data (77.2%). However, this difference is not significant (*p* > 0.05). Similarly, the LDA classifiers trained with the combined TD features and the arm position information exhibited the same classification accuracy (84.3%) for both the discrete and continuous position training.

The SVM classifiers also showed a similar performance difference. The TD feature-based classifier for the discrete position data showed a slightly better but insignificant (*p* > 0.05) classifier accuracy (93.4%) as compared to the continuous motion data (92.9%). Similarly, the SVM classifier trained with the TD-POS features of the discrete position data resulted in an average classification accuracy of 97.6%, which is significantly (*p* < 0.05) <98.7% for the classifier trained with same features of the dynamic forearm movement data. The results of the classifiers prove that only the SVM classifier trained with the TD-POS features shows a significant difference between the static position and dynamic forearm movement-based training approach. It is interesting to note that although the performance difference between the two training approaches is significant for only the SVM classifier, the continuous position training, which involves a simple movement of the forearm along two trajectories, is certainly a more realistic and pragmatic approach as compared to the discrete position training, which requires expert supervision and is physically more demanding. The simplicity of the dynamic movement training approach is also significant since classifier training on a daily basis is necessary for consistent classifier performance (Amsuss et al., 2013) and, thus, reliable prostheses control.

**Figure 10:**
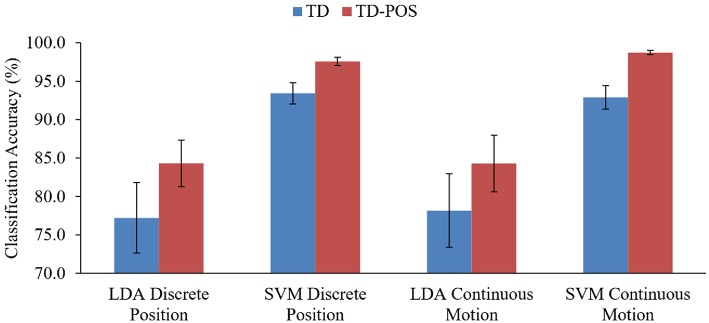
Performance comparisons of TD and TD-POS features-based classifiers. Results are averaged across all subjects and classes. A significant performance improvement is shown by all the position aware classifiers.

A significant effect of arm position was observed on classifier performance (Figure 10). The LDA classifier trained with the TD-POS features showed an improvement of 7.1% (*p* < 0.05) for the discrete position data and an improvement of 6.1% (*p* < 0.05) for the continuous motion data as compared to classifiers trained with only the TD features. The position-aware SVM classifiers also showed a significant improvement of 4.1% (*p* < 0.05) for the discrete position data and an average improvement of 5.8% (*p* < 0.05) for the continuous movement data. These results show a substantial effect of limb position on classifier performance for both the discrete and continuous position training approach. Previous studies by Scheme et al. (2010), Jiang et al. (2013), Radmand et al. (2014), and Yu et al. (2018) also showed a significant dependence of classifier accuracy on positional variation. Gravitational effects, changes in muscle length, and moment arm are the possible reasons for this variation. The extra muscular effort while moving and stabilizing the forearm against gravity certainly causes the recruitment of additional muscle fibers, causing a variation in muscular activity.

**Figure 11:**
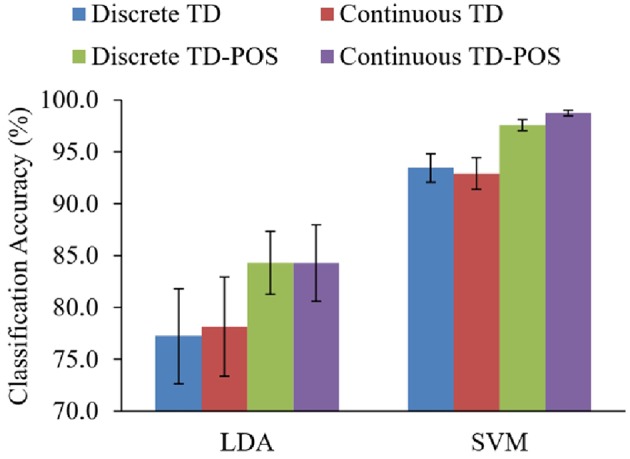
Comparisons of LDA and SVM classifiers. Results are averaged across all subjects and classes.

**Figure 12:**
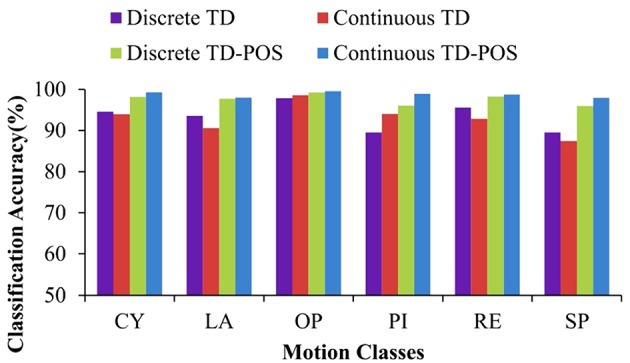
Classifier accuracy across individual classes. Performance of the SVM classifiers for each motion class averaged across all subjects.

**Figure 13:**
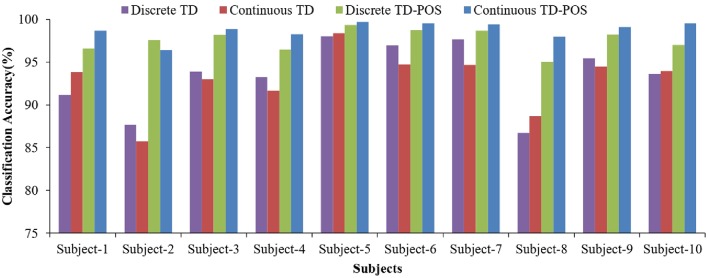
Classifier accuracy across individual subjects. Results indicate the performance of the four SVM classifiers averaged across all motion classes for individual subjects.

The SVM classifier performed significantly better than the LDA classifier. The performance difference is consistent for all the training and testing scenarios (Figure 11) with an average difference of 16.2, 13.3, 14.7, and 14.4% for the discrete position TD, discrete position TD-POS, continuous position TD, and continuous position TD-POS-based classifiers, respectively. The improvement in performance can be attributed to improved class separability due to non-linear decision boundaries of the SVM classifier. An intercomparison of the SVM classifiers shows the continuous position classifier performing significantly (*p* < 0.05) better than the discrete position classifier. This improvement is consistent across all the motion classes (Figure 12) and subjects (Figure 13). The OP class was most accurately classified by all the four SVM classifiers. A possible reason is the taxonomical difference of this class (all the fingers extended) from the rest of the classes. Across individual subjects, the best classification accuracy for all the SVM classifiers was achieved for subject 5. The classifier performance variation across subjects is expected as it is a known fact that sEMG signals for the same gestures are different for different subjects. Moreover, the physiological properties of muscles and, hence, the sEMG signals also depend on the nature of the daily life activities, which may differ across subjects. It can be concluded from the results that the continuous position SVM classifier outperforms other classifiers for all the different training and testing scenarios.

The TD-POS features-based LDA classifier, trained with the discrete position data, resulted in an average accuracy of 84.3 ± 3.0%, which is lower than 95% reported by Fougner et al. (2011) using five static training and testing positions with an LDA classifier. This difference in performance can be attributed to the reduced number of sEMG channels (four sEMG and two IMUs) as compared with the eight channels of the sEMG and two accelerometers used in the previous study. Another study, by Geng et al. (2012), also reported a classification accuracy of 92.7% for a six-class problem when using five static testing and training positions with eight channels of sEMG and two channels of ACC-MMG. Another probable reason for the comparatively low performance is the different set of motion classes used in this study. Although it is a known fact that opposite motion classes (e.g., pronation and supination, OP, and close) are easier to distinguish as compared to classes that are taxonomically more similar, the sensitivity of classifier accuracy to different sets of motion classes needs further investigation.

**Figure 14:**
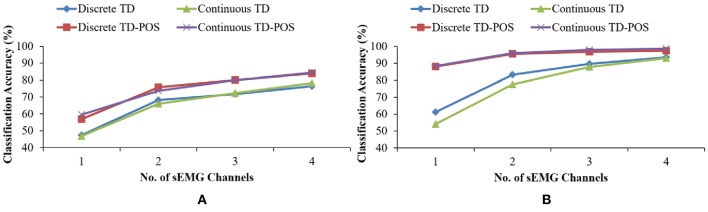
Classification accuracy variations for LDA **(A)** and SVM **(B)** classifiers as the number of sEMG channels are increased from 1 to 4.

The improved performance of the position-aware LDA and SVM classifiers advocates the use of sensor fusion techniques for classifier robustness against positional variation. Another advantage of sensor fusion is evident from Figure 14, which shows the classifier performance variation as the number of electrodes is increased from 1 to 4. The TD features-only SVM classifiers for both the discrete and continuous position case require at least four electrodes for achieving more than 90% accuracy, while the TD-POS classifiers achieve more than 95% accuracy with only two sEMG channels. Although it can be argued that the position-aware classifier also utilizes four channels of information (two channels of sEMG and two IMUs) and has no significant advantage over the four-channel sEMG classifier, it is a known fact that integrating the inertial sensors in prostheses is technically less challenging as compared to integrating sEMG electrodes. Moreover, the inertial sensors are less prone to noise and other factors as compared to the sEMG sensors. Thus, sensor fusion techniques can reduce the complexity and improve the reliability of existing prosthetic devices.

## Conclusions

In this research, hand motion classifier performance under static and dynamic training scenarios was studied, and the adverse effect of the arm position variation on classifier performance was also evaluated. Results have shown no significant performance difference for the LDA classifiers for both training strategies; however, the SVM classifiers showed significantly improved performance for the dynamic training as compared to the discrete position training. This is a significant finding since the continuous position training, which involves a simple movement of the forearm along a trajectory, neither requires expert supervision and lengthy training sessions nor does it need specialized setup for training at specified discrete positions. The dynamic training is certainly a more pragmatic approach from the end user point of view, and the authors believe that improvement in training procedures can certainly improve the reliability and acceptability of active prosthetic devices. Results have also shown a profound impact of arm position on classifier accuracy and have proved the effectiveness of sensor fusion techniques to improve classifier performance and reliability.

## Ethics Statement

Each subject signed a written consent form in accordance with the Declaration of Helsinki duly approved by the ethical committee of the institution.

## Author Contributions

WS, YA, and MJK designed the study. WS designed the hardware and software, performed the experiments, and drafted the paper. MJK and NN formally analyzed the data and interpreted the results. MK critically reviewed the drafted paper. All the authors read the manuscript and consented for its publication.

### Conflict of Interest Statement

The authors declare that the research was conducted in the absence of any commercial or financial relationships that could be construed as a potential conflict of interest.
