# Supplementary figures and images for: Enhanced Performance for Multi-Forearm Movement Decoding Using Hybrid IMU–sEMG Interface
Source: Front Neurorobot. 2019 Jul 3;13:43. doi: 10.3389/fnbot.2019.00043 (PMC6617522; doi:10.3389/fnbot.2019.00043)

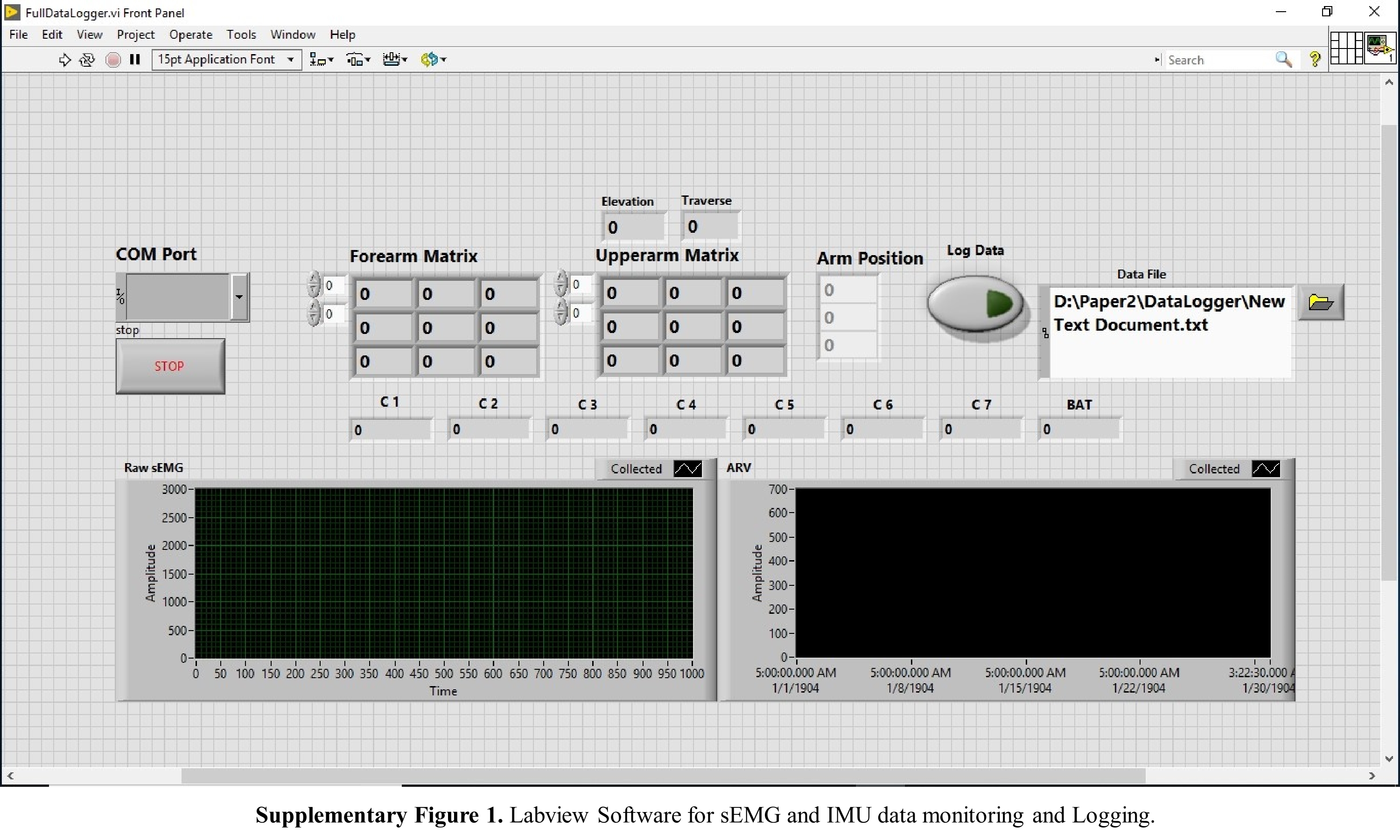

Supplement: Supplementary file 1 [file Image_1.jpg]

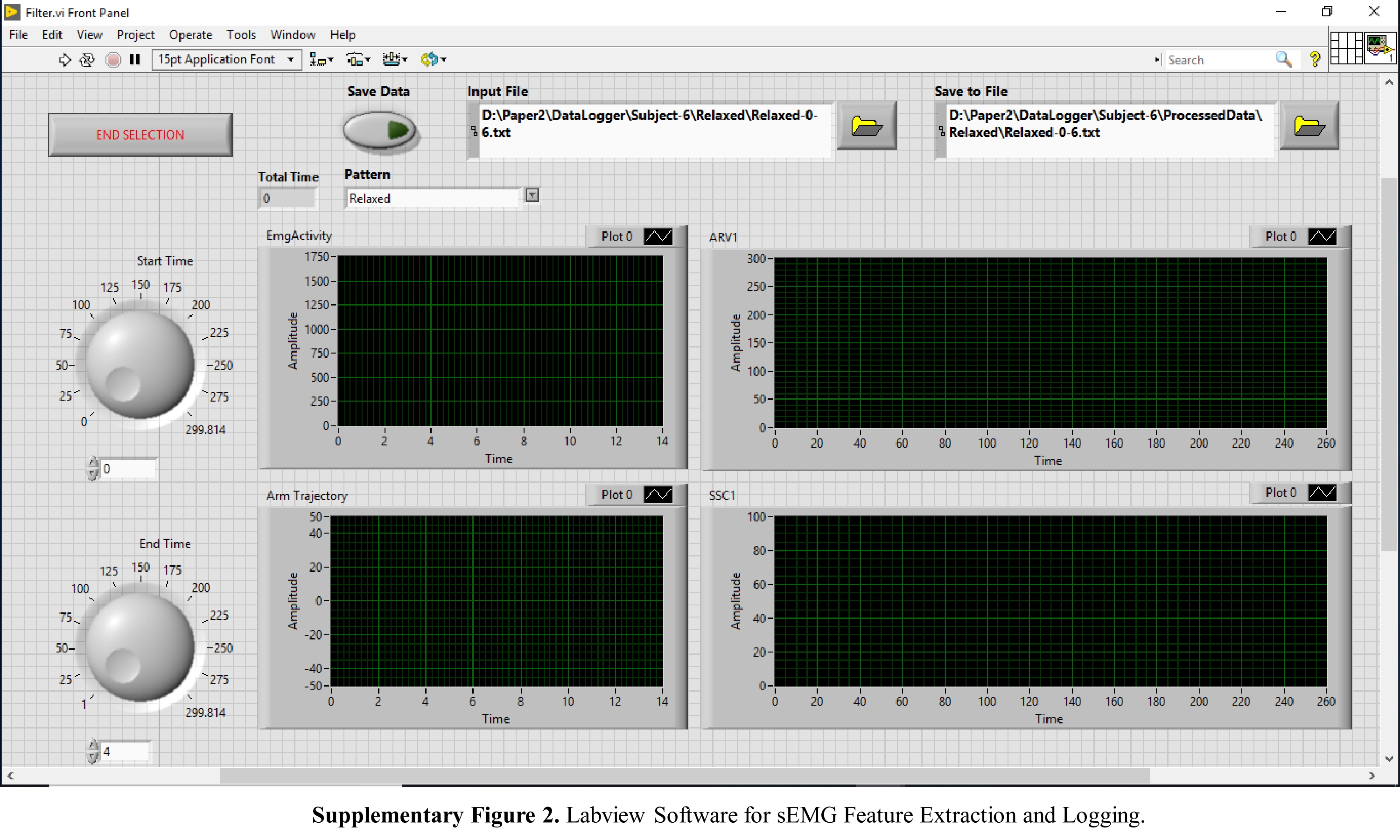

Supplement: Supplementary file 2 [file Image_2.jpg]
