# Supplementary material for: Enhanced Performance for Multi-Forearm Movement Decoding Using Hybrid IMU–sEMG Interface
Source: Front Neurorobot. 2019 Jul 3;13:43. doi: 10.3389/fnbot.2019.00043 (PMC6617522; doi:10.3389/fnbot.2019.00043)
Supplement: Supplementary file 3 [file Image_3.pdf]

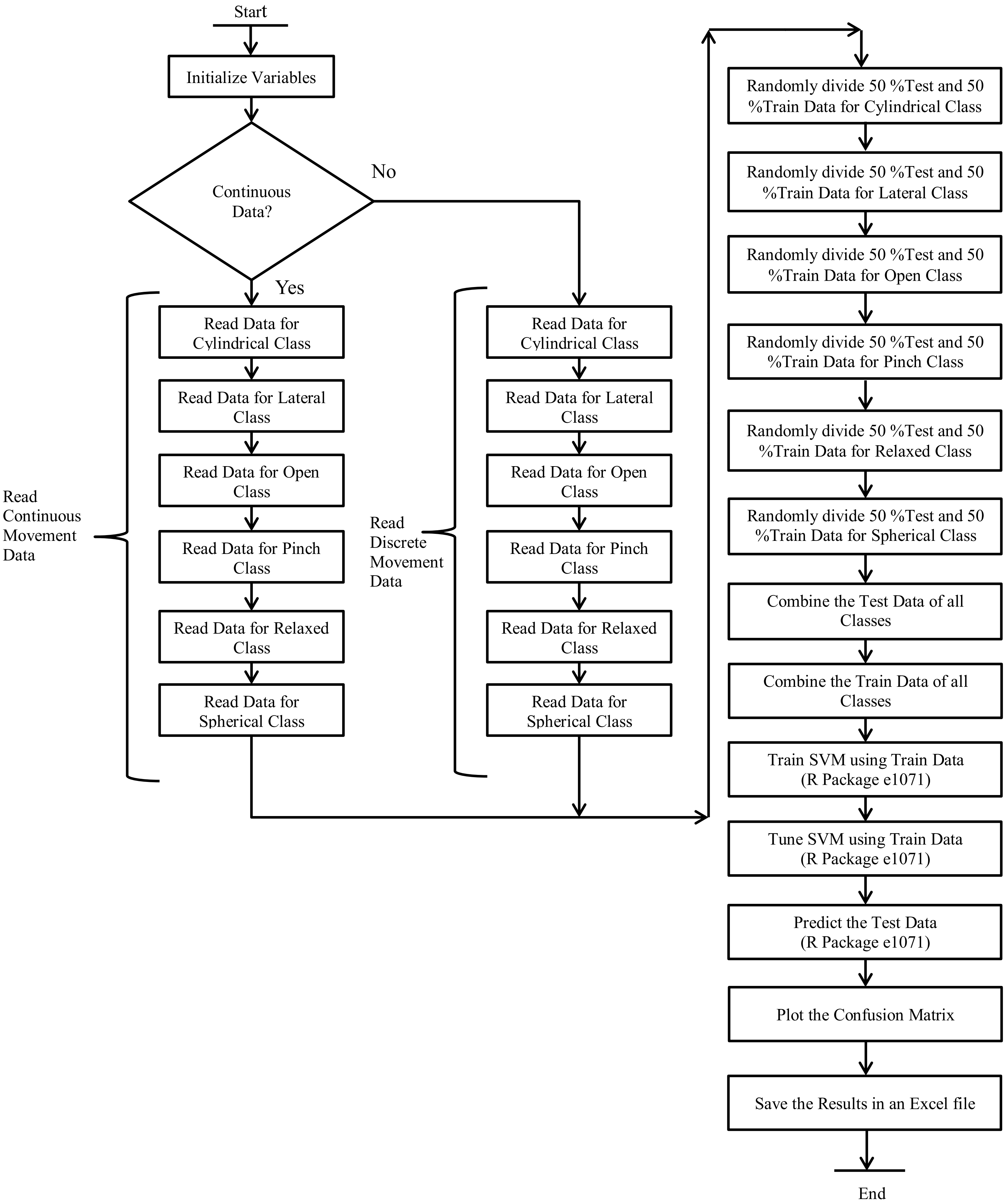


**Supplementary Figure 3.** Flow diagram for training and testing of the SVM classifier for both the discrete and continuous position data.
